# CRB2 completes a fully expressed Crumbs complex in the Retinal Pigment Epithelium

**DOI:** 10.1038/srep14504

**Published:** 2015-09-25

**Authors:** Antonio E. Paniagua, Saúl Herranz-Martín, David Jimeno, Ángela M. Jimeno, Saray López-Benito, Juan Carlos Arévalo, Almudena Velasco, José Aijón, Concepción Lillo

**Affiliations:** 1 Institute of Neurosciences of Castilla y León, IBSAL, Cell Biology and Pathology, University of Salamanca, 37007, Salamanca, Spain

## Abstract

The CRB proteins CRB1, CRB2 and CRB3 are members of the cell polarity complex Crumbs in mammals that together with Scribble and Par complexes stablish the polarity of a variety of cell types. Although many members of the Crumbs complex proteins are expressed in the retinal pigment epithelium (RPE), and even though the mRNA of CRB2 has been detected in ARPE-19 cells and in the RPE/Choroid, to date no CRB protein has yet been found in this tissue. To investigate this possibility, we generated an antibody that specifically recognize the mouse CRB2 protein, and we demonstrate the expression of CRB2 in mouse RPE. Confocal analysis shows that CRB2 is restricted to the apicolateral membrane of RPE cells, and more precisely, in the tight junctions. Our study identified CRB2 as the member of the CRB protein family that is present together with the rest of the components of the Crumbs complex in the RPE apico-lateral cell membrane. Considering that the functions of CRB proteins are decisive in the establishment and maintenance of cell-cell junctions in several epithelial-derived cell types, we believe that these findings are a relevant starting point for unraveling the functions that CRB2 might perform in the RPE.

Cell polarity is a known property of cells from unicellular and multicellular organisms, defined as an asymmetry in cell shape, protein distribution and cell function^1^. In multicellular organisms, cell polarization is essential both in the embryonic stage and adulthood for the proper performance of a wide range of cellular processes as diverse as synaptic communication between neurons^2^,^3^, the formation of epithelial barriers to maintain tissue homeostasis^4^ and the proper distribution of cellular components during cell division^1^. Therefore, cell polarity must be a highly controlled process, which is achieved by cell-surface landmarks that adapt core pathways for the correct assembly of the cytoskeleton and protein transport. These cell-surface landmarks are thought to be composed of a few evolutionary conserved proteins included in three polarity protein complexes named the Scribble, Par and Crumbs complexes^1^,^3^,^5^,^6^,^7^.

The Crumbs gene was initially identified in Drosophila, where it encodes a transmembrane protein that determines apicobasal polarity in embryonic epithelial cells^8^ and controls the integrity of adherens junctions in the adult fly eye^9^. Drosophila Crumbs contains a large extracellular domain, a transmembrane portion and a short intracellular portion^8^,^10^,^11^,^12^, which acts as an anchoring point for the assembly of the intracellular Crumbs complex^10^,^13^,^14^. The Crumbs complex is typically composed by PALS1, PATJ and the Crumbs homologs (CRB) proteins^15^,^16^; and plays a crucial role in apical domain specification of the plasma membrane^3^,^8^,^17^. To date, CRB proteins have been identified in several organisms, ranging from invertebrates to mammals, showing that they are highly evolutionary conserved proteins^10^,^15^,^18^,^19^. In mammals, the CRB family is composed of three members: CRB1, CRB2 and CRB3^20^,^21^,^22^. All three CRB proteins share the highly conserved intracellular domain, and whereas CRB1 and CRB2 have a large extracellular component CRB3 lacks this part^15^,^20^,^21^,^22^. CRB proteins have been described to be essential for many cellular processes as the early embryonic development^23^, formation and maintenance of tight junctions^24^,^25^,^26^ or cell division and ciliogenesis^27^,^28^,^29^. CRB1 has been extensively investigated because CRB1 mutations cause several human retinal dystrophies, such as Leber congenital amaurosis or type 12 Retinitis Pigmentosa^22^,^30^,^31^. In the mouse retina CRB2 is also essential for the correct expression and location of the adherens junctions proteins and removal of mouse *Crb2* from retinal progenitor cells shows abnormal lamination of the neuroepithelial layer during development and a progressive thinning of the photoreceptor layer in adulthood^32^,^33^.

The Retinal Pigment Epithelium (RPE) is a highly polarized tissue that is essential for the maintenance of retinal homeostasis^34^,^35^. As a polarized epithelium, it expresses some of the Crumbs complex members such as MPP1, PATJ, EPB41L5 and PALS1^36^,^37^,^38^,^39^ but, unless CRB proteins are necessary for the anchorage of the Crumbs complex to the cell membrane^7^, none of them have been detected in this tissue so far. To investigate the expression of one of the members of the CRB protein family in this tissue, we designed and generated an antibody that specifically recognize the mouse CRB2 protein inasmuch as, to date, all antibodies used in previous studies have failed to fully elucidate the expression of this protein in this tissue.

## Results

### Analysis of the expression pattern of the *Crb2* and *Crb3* genes

**Figure 1:**
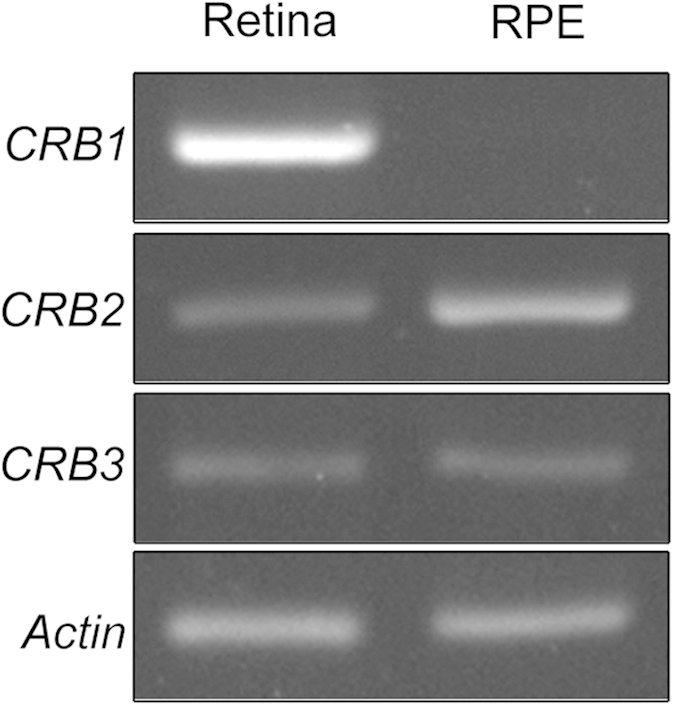
*Crb1, Crb2, Crb3* and *Actin* mRNA amplification in retina and RPE by RT-PCR assays. *Crb2* and *Crb3* are transcribed into mRNA in both retina and RPE while *Crb1* mRNA is transcribed in retina but not in the RPE. *Actin* amplification levels are similar in both tissues.

The expression pattern of the three *Crb* genes (*Crb1*, *Crb2* and *Crb3*) was determined by reverse transcription (RT)-PCR on mRNA samples from mouse retina and RPE. The three transcripts are present in the retina as expected but we also found that out of the three, CRB2 and CRB3 are also expressed in the RPE (Fig. 1).

### Specificity of the CRB2 antibody

To accomplish the present study, we designed a new antibody to specifically recognize and discriminate the CRB2 protein. Our goal was to try to improve the detection pattern and disparities both by immunofluorescence (IF) and western blot (WB) techniques, shown in previous works that used different sets of custom-made antibodies that recognized diverse parts of the extracellular domains^33^,^36^,^40^,^41^,^42^,^43^.

**Figure 2:**
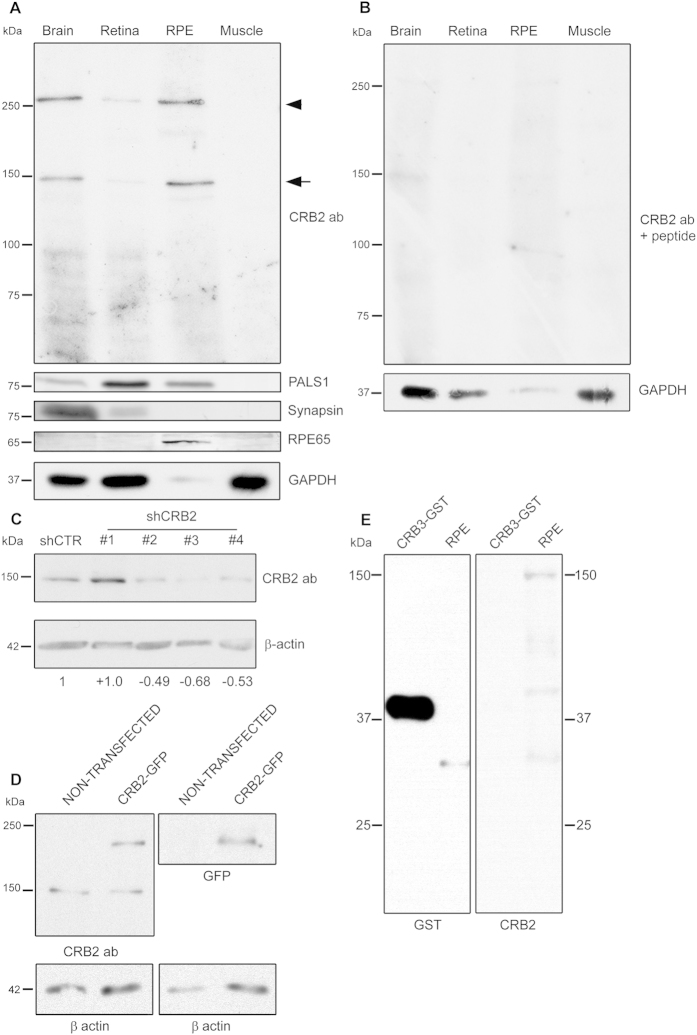
Specificity analyses for the CRB2 antibody and CRB2 expression in the RPE. (**A**) The CRB2 antibody detects two bands with approximate molecular weights of 150 kDa (arrow) and 260 kDa (arrowhead) in brain, retina and RPE, but not in muscle. A member of the Crumbs complex, PALS1, is also expressed in the same tissues. The synapsin protein, only present in brain and retina lysates, and the expression of RPE65, only found in RPE, rule out any cross-contamination between retinal and RPE lysates. (**B**) In the peptide competition assay, the two bands detected at 150 and 260 kDa with the CRB2 antibody disappear. GAPDH was used as loading control. (**C**) CRB2 antibody detects a band of approximately 150 kDa in N2A cells. The intensity of this band decreases in cells transfected with three different shRNAs of a set of four (shCRB2) when compared with shRNA control (shCTR) transfected cells. The numbers indicate the CRB2 increase/decrease values relative to the shCTR. (**D**) CRB2-GFP fused protein overexpressed in HEK293 cells (CRB2-GFP) is detected as a band of 180 kDa by CRB2 and GFP antibodies but not in non-transfected HEK293cells. The 150 kDa band is also detected by the CRB2 antibody in both transfected and non-transfected HEK293 cells. β-actin was used as loading control. (**E**) CRB3 protein fused to GST is detected wit GST antibody at 40 kDa but not with CRB2 antibody. RPE tissue was used as a control of CRB2 antibody viability.

The specificity of the CRB2 antibody was determined by WB assays. Total protein extracts from brain and retina, where the presence of CRB2 has been described were employed as positive controls for the expression of CRB2^36^,^40^. Proteins obtained from skeletal muscle, where CRB2 mRNA has not been detected^21^, were used as a negative control. The CRB2 antibody detected two bands in brain and retina extracts, one with an approximate molecular weight of 150 kDa (arrow in Fig. 2A) and another one of approximately 260 kDa (arrowhead in Fig. 2A). These two bands were absent in the muscle protein extract (Fig. 2A). Furthermore, the same bands were also identified in the RPE protein extract (Fig. 2A). To rule out any possible cross-contamination between the RPE and retinal tissues, the synapsin protein was chosen as a control marker for nervous tissue and RPE65 was selected as an RPE-specific indicator (Fig. 2A). In addition, we used PALS1 to test the expression of a member of the Crumbs complex, which we found in brain, retina and the RPE but not in muscle (Fig. 2A). Different methods were employed to verify the specificity of the CRB2 antibody. First, we carried out a peptide competition assay, observing the almost complete disappearance of the two bands in the control tissues and in the RPE (Fig. 2B). We then knocked down endogenous CRB2 in Neuro-2A (N2A) cells by transfecting them with four different shRNA sequences against Crb2 mRNA. Three of the four sequences showed a great effectivity knocking down Crb2, since the intensity of the 150 kDa band detected in N2A lysates decreased a 49,06% ± 7,19 for shCRB2#2, a 68,71 % ± 3,90 for shCRB2#3 and a 53,71% ± 14,39 for shCRB2#4 (Fig. 2C). We also overexpressed the CRB2 protein fused to GFP in Human embryonic kidney (HEK) 293 cells. The WB analysis revealed the exogenous protein at an approximate molecular weight of 180 kDa in the transfected cells using both the CRB2 antibody and the GFP antibody (Fig. 2D) and the endogenous CRB2 protein at 150 kDa in both transfected and non-transfected HEK293 cells (Fig. 2D). Finally, we wanted to discard the possibility that the CRB2 antibody may recognize the CRB3 protein due to aminoacidic similarities. To do this, we loaded a commercial CRB3 protein fused to GST and performed immunoblotting with the CRB2 antibody. The CRB2 antibody did not recognize the CRB3-GST fusion protein, which was effectively detected with the GST antibody (Fig. 2E). We then discarded the possibility that the CRB2 antibody may cross-react with the CRB3 protein.

Therefore, the CRB2 antibody specifically detects CRB2 at least at 150 kDa that confirms the expression of CRB2 in mouse RPE cells by WB.

### CRB2 is located apically to the proteins of the adherens junctions in the RPE

**Figure 3:**
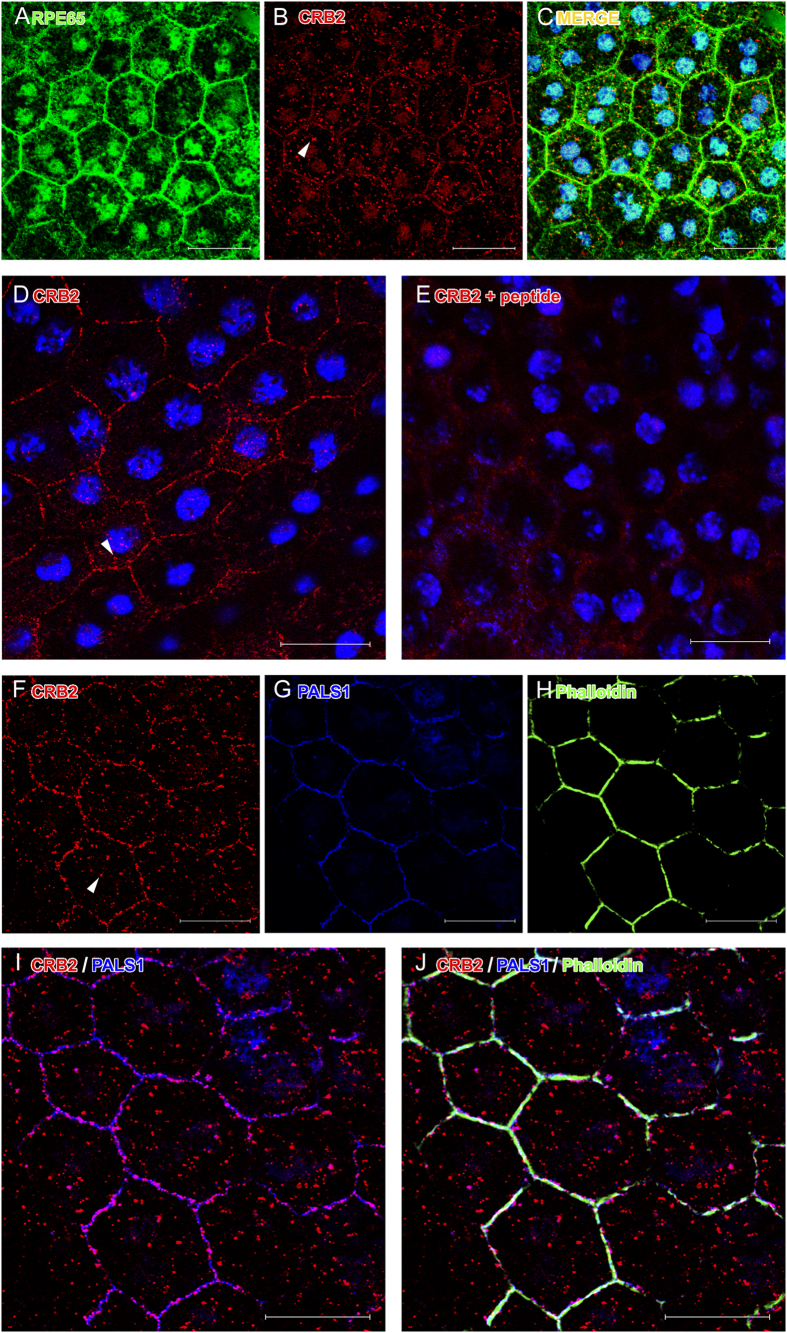
CRB2 detection by IF on RPE flatmounts. RPE65 (**A**) is expressed in RPE cells together with CRB2 (**B**). (**C**) Merged images of RPE65 and CRB2 labeling and nuclei in blue. (**D**) CRB2 is located in the cell membrane of RPE cells. (**E**) The CRB2 labeling disappears after the peptide competition assay. Nuclei are stained in blue in (**D**,**E**). Triple IF showing CRB2 (**F**), PALS1 (**G**) and phalloidin (**H**) in the cell membrane. CRB2 and PALS1 colocalize in the cell membrane (**I**) together with phalloidin (**J**). Arrowheads in (**B**,**D**,**F**): scattered punctate labeling for CRB2 in the cytoplasm. Scale bars: 20 μm.

In order to determine the localization of CRB2 in the RPE, we performed detailed confocal imaging analyses in RPE flatmounts. The co-immunolabeling with RPE65 (Fig. 3A), a marker specific for RPE cells, and the CRB2 antibody (Fig. 3B) showed that CRB2 was mostly located in the plasma membrane of the RPE cells (Fig. 3C), although we also found a faint, scattered labeling in the cytoplasm (arrowheads in Fig. 3B,D,F). All this labeling disappeared with the peptide competition assay (Fig. 3E).

**Figure 4:**
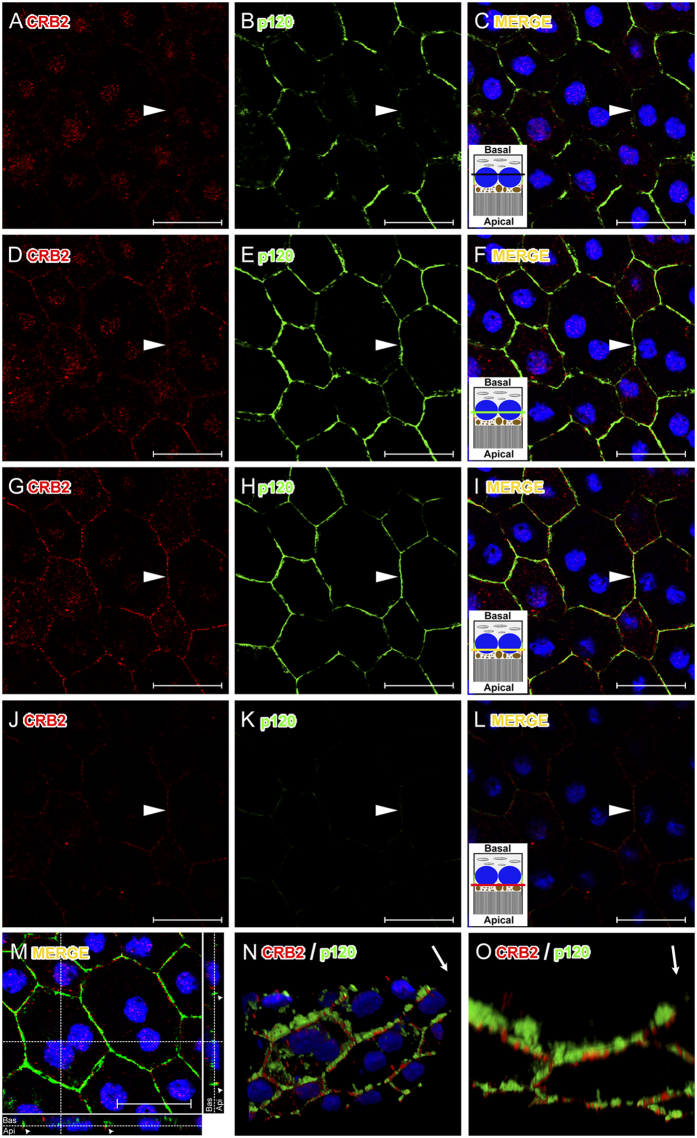
Accurate location of CRB2 in the cell membrane of RPE cells. (**A**–**L**) Confocal microscopy Z-stack images of a triple immunolabeling for CRB2 (red), p120 catenin (green) and nuclei (blue) in RPE flatmounts obtained from the basal to apical cell sides with a step size of 300 nm. In the lateral membrane, p120 catenin is expressed more basally (**B**,**E**,**H**) and CRB2 is located apically (**G**,**J**). None of these proteins is located at the basal side of the cell membrane (arrowheads in **A**–**C**). Towards the apical side, p120 catenin appears (arrowhead in **E**,**F**), but not CRB2 (arrowhead in **D**). Both proteins colocalize in the cell membrane across a short range (arrowheads in **G**–**I**). CRB2, but not p120 catenin, is localized at the apical-most side of the cell membrane analyzed (arrowheads in **J**–**L**). In (**C**,**F**,**I**,**L**) an RPE cell scheme represents the relative position where each image was acquired. The black line in C represents the portion of the RPE where none of the proteins studied are located. The green line in F is where only p120 catenin is located. The yellow line in (**I**) is where both proteins (p120 catenin, green and CRB2, red) colocalize. The red line in (**L**) represents the area of the RPE cell where CRB2, but not p120 catenin, is present. (**M**) maximal projection and orthogonal view of the Z-stacks shows the location of the two proteins (arrowheads) in the apical side of the cells. (**N**) a three-dimensional reconstruction of the Z-stack shows that CRB2 is located apically to p120 catenin. (**O**) intracellular staining was removed to facilitate the examination of the membrane labeling. Arrows in the upper right margin in (**N**,**O**) indicate the orientation from basal (base of the arrow) to apical side of the cells (arrowhead). A movie of this 3D reconstruction is available on “Video file”. Scale bars: 20 μm.

We then investigated the precise location of CRB2 in the RPE plasma membrane. To do this, we carried out double and triple IF experiments in RPE flatmounts for CRB2 and several apical membrane and cell junctions markers present in these cells, such as PALS1 (Fig. 3G), phalloidin (Fig. 3H), which labels the F-actin associated with these junctions and p120 catenin (Fig. 4). The triple IF experiments revealed that phalloidin, PALS1 and CRB2 colocalized, all of them being detected in the same area of the RPE plasma membrane (Fig. 3F–J). Nevertheless, the CRB2 protein showed a more restricted and scattered distribution in the plasma membrane than the other proteins, which delineated the entire perimeter of the cell junctions’ area.

To obtain a more detailed visualization of the exact location of CRB2 in the apicolateral membrane of RPE cells, we analyzed the three-dimensional distribution of this protein, together with some cell adhesion proteins, such as p120 catenin (Fig. 4), ZO1 and phalloidin (unpublished data). To accomplish this, we obtained Z-stack images by confocal microscopy, and found p120 catenin located in the apicolateral membrane (Fig. 4B,E,H,K) while CRB2 was located in a narrower range apically to p120 catenin (Fig. 4A,D,G,J). These proteins did colocalize, but in a very specific area (Fig. 4G–I). Likewise, the orthogonal view of the Z-stack (Fig. 4M) and the three-dimensional reconstruction performed with these images to pinpoint the location of the two proteins demonstrated that CRB2 was positioned apically to p120 catenin in the apicolateral membrane of RPE cells (Fig. 4N,O and Video file).

## Discussion

As a polarized epithelium, the RPE expresses some of the Crumbs complex proteins^20^,^36^,^37^,^38^,^39^,^40^, although, to date, none of the three CRB protein family members had been detected. Given the relevance that CRB proteins have shown in other cells or tissues^22^,^23^,^44^ and the importance of polarity for the RPE functions^45^,^46^, the aim of this study was to unravel whether CRB2 is expressed in the mouse RPE and to determine its precise localization in this tissue.

In the present work, we have demonstrated the presence of the mRNA of *Crb2* in the mouse RPE and confirmed the absence of *Crb1* mRNA, in agreement with a previous work where *Crb2* mRNA was detected in RPE/Choroid and ARPE19^21^. Additionally, the presence of *Crb3* mRNA in the RPE observed in the present study had not been described in previous studies. However, subsequent studies have not been able to detect the expression of any of these proteins in the RPE. One of the reasons that could explain this circumstance is that the specific conditions used in the detection protocols when using these antibodies were not the most suitable for the antibodies and techniques that were used. Many of these antibodies are directed against the extracellular domain of CRB2, none of them have been commercialized so far or have been designed to recognize the human CRB2 protein^33^,^36^,^40^,^41^,^42^,^43^. Furthermore, there is substantial controversy regarding the precise molecular weight of this protein, since these publications either do not show any WB analysis, or in the case they do, these studies do not agree in conferring a certain molecular weight to CRB2 or show many bands of diverse molecular weight in different tissues^40^,^41^,^42^,^47^. To try to solve these inconsistencies and to elucidate the presence of CRB2 in the mouse RPE, we designed and characterized a specific antibody against the cytoplasmic domain of mouse CRB2.

Our WB data support the expression of the CRB2 protein in the RPE, as well as in the brain and retina, where it had been already detected^33^,^36^,^40^. Even though the predicted molecular weight for CRB2 is 134 kDa (Universal Protein Resource), we have detected two bands in these tissues, one of them slightly under 150 kDa and the other one above 260 kDa. These results are partly consistent with those reported in a previous study in which CRB2 was detected as three bands of 80, 150 and 200–220 kDa in mouse embryonic stem cells^42^ and with an additional band of 220 kDa in forebrain lysates of mouse embryos of 13.5 days^40^. The overexpression of CRB2 fused to GFP in HEK293 showed the exogenous protein at 180 kDa that was detected with both, CRB2 and GFP antibodies. The endogenous 150 kDa band was clearly identified in the two cell lines used for the different experiments in the present study, which was effectively knocked down in the N2A cells, confirming the specificity of the CRB2 antibody. Despite the fact that this antibody was generated to detect the mouse protein, it seems that it also distinguish the human CRB2 in HEK293 cells, probably because of the high similarity of their cytoplasmic domains. However, we could not detect the heaviest 260 kDa band in any of the cell lines employed in our study, becoming more difficult to confirm the specificity of the 260 kDa band detected in tissue lysates. The complexity of the cell organization required in a tissue differs from that of a cell culture, and that could explain the presence of different isoforms, post-translational modifications or even protein aggregates in tissue protein extracts. Further studies are needed to confirm this observation.

Regarding the detection of the CRB2 protein in tissue, although previous studies have shown the expression and location of CRB2 in retina sections that include the RPE cells tissue layer^32^,^33^,^36^,^48^, none of them has reported the expression of this protein in the RPE. This could be due to the conditions of the procedures, as the tissue processing (e.g., retina cryosections, RPE flatmounts), the fixation time, the fixative concentration or the antibodies used, since these conditions are critical for the preservation and detection of many antigens. In fact, a clear example of the convenience of using the appropriate tissue processing is that we were not able to detect CRB2 in RPE cells in tissue cryosections (data not shown) but only in RPE flatmounts. Interestingly, this observation is not unusual since one of the Crumbs complex proteins, PALS1, was not detected in the RPE in retina sections either^38^,^49^,^50^,^51^ but only using the RPE flatmounts procedure^38^. The detailed confocal microscopy analysis of RPE flatmounts for CRB2 and proteins such as p120 catenin and PALS1 and 3D reconstruction analyses allowed us to discern that CRB2 is located in a narrow area situated apically to the zone where the adherens junction are located, what is in agreement with the location of Crumbs complex proteins in other cells^25^,^52^,^53^,^54^.

The Crumbs complex protein PALS1 has been described to be essential for the RPE and retina structure maintenance^38^,^55^. Interestingly, we found that the staining of CRB2 and PALS1 did not completely overlap, since CRB2 showed a patchy distribution in the RPE cell membrane, differing from the more continuous PALS1 staining throughout it. These data suggest that in the RPE cell membrane PALS1 may not always be bound to CRB2. We have demonstrated that *Crb3* mRNA is expressed in these cells so it is possible that CRB3 protein could be also expressed in the RPE cells since it has been detected in several epithelial cell lines^20^,^24^,^44^,^54^,^56^. The co-expresion of both members of the CRB family in the same cell has already been reported^15^,^49^ and although both proteins are very similar, CRB3 (28 kDa), which contains a shorter extracellular portion^20^ is involved in many diverse processes, as cell division or ciliogenesis in other epithelial cells^27^,^29^. This way, the expression of another member of the CRB protein family is still unexplored and needs to be elucidated to better understand the mechanisms in which these two CRB proteins might be implicated.

Our IF experiments in RPE flatmounts also show a scattered and punctate cytoplasmic labeling for CRB2 on these cells. Previous studies in Drosophila reported that an active traffic between the plasma membrane and cytoplasmic vesicles is necessary for the control of Crumbs protein function^57^,^58^. In mammalian MDCKII (Madin-Darby Canine Kidney II) cells a noteworthy trafficking of CRB3 protein has recently been demonstrated, where an important amount of this protein has been detected in the cytoplasm as well as in the apical cell membrane^56^. Our results suggest that the punctate labeling obtained for CRB2 in the cytoplasm of RPE cells could correspond to the different spots of localization of the protein while travelling from or towards the membrane, although further studies are needed to prove this observation.

We have tried to confirm the specificity of the CRB2 immunolabeling in IF experiments by knocking down this protein from N2A cells. Unfortunately, maybe due to the incomplete knock down by using this procedure together with the fact that IF is mainly qualitative and not quantitative enough, we have not been able to get definitive conclusions out of the IF experiments after knocking down CRB2 from N2A cells. However, both the precise location of CRB2 in RPE cells that is in agreement with the location of the Crumbs complex proteins in epithelial cells^20^,^25^,^52^,^53^,^54^ and all the experiments performed by WB showing the distinct expression of CRB2, strongly support the specificity of the IF labeling for CRB2 in RPE cells.

In conclusion, our findings are relevant because this is the first time that any of the CRB proteins have been found in RPE. To date, RPE cells were an exception, since it is a cell type where some members of the Crumbs complex proteins but none of the CRB proteins had been detected. This way we have demonstrated that, at least in the RPE, a fully Crumbs complex is expressed, probably being CRB2 the protein that anchors the polarity complex to the cell membrane. In light of this, considering the importance of proper RPE cell adhesion organization and polarization for the maintenance of retinal homeostasis, and bearing in mind the retinal disorders caused by the improper functioning of Crumbs complex in these tissues, we believe that these findings are a relevant starting point for gaining further insight into a possible role of these proteins in the correct performance of the functions of the RPE.

## Materials and Methods

All procedures used in this work were in accordance with the guidelines of the European Communities Council Directive 2010/63/UE and the RD 53/2013 Spanish legislation for the use and care of animals. All the details of the study were approved by the Bioethics Committee of the University of Salamanca.

### Animals

For this study, we used 90-day-old adult wild-type C57BL/6J mice that were euthanized with carbon dioxide prior to tissue extraction.

### RT-PCR analyses

To verify *Crb1*, *Crb2* and *Crb3 and Actin* mRNA expression, four pairs of primers were designed from the corresponding gene sequences and used for RT-PCR analyses using mouse retina and RPE cDNA. *Crb1* (exon 6): Forward 5′-ACAAGTGGCTCCCATACAGG-3′ and Reverse 5′-ACGAAATGCCATTCTCCATC-3′, *Crb2* (exons 2–4): Forward 5′-GCAGATCACTACGAGTGCC-3′ and Reverse 5′-CCGGAAACCGTTGACCAG-3′, *Crb3* (exons 2–4): Forward 5′-TAACAGCACCGGACCC-3′ and Reverse 5′-ACTCCCAGAATGGAAAAGAC-3′, *Actin*: Forward 5′-AGCCATGTACGTAGCCATCC-3′ and Reverse 5′-ACCCTCATAGATGGGCACAG3′. After the animals had been sacrificed, the eyes were dissected out, the cornea and lens were removed, the retina was extracted and the RPE was separated from the choroid using fine forceps. The retina and the RPE were lysed in TRIzol® LS Reagent (Life Technologies^TM^) and total RNA was isolated following the manufacturer’s instructions. Total RNA was treated with TURBO DNA-free^TM^ kit (Life Technologies^TM^) to digest trace amounts of genomic DNA. Randomly primed cDNA synthesis was performed using the RETROscript® kit (Life Technologies^TM^) in accordance with the manufacturer’s instructions. Specific sequence amplifications of the *Crb1*, *Crb2, Crb3 and Actin* genes were performed with PCR using 300 μg of cDNA and 0.2 μM to 0.5 μM of specific primers in 20 μl of PCR solution under the following conditions: an initial denaturation period of 3 minutes at 94 °C followed by an amplification period of 35 cycles for 45 seconds at 94 °C, 30 seconds for annealing at 49 °C in the case of *Crb1* pair of primers and 56 °C for *Crb2, Crb3 and Actin* pairs of primers and 30 seconds at 72 °C, and a final extension timing of 10 minutes at 72 °C.

To analyze the RT-PCR reaction, 20 μl of each product was loaded onto a 2% agarose gel in the presence of ethidium bromide and visualized under a UV light transiluminator.

Negative controls such as minus-RT and a minus-template PCR ruled out any DNA contamination of the RNA samples or the PCR reagents.

### Cell cultures and transfection

N2A cells were cultured in Dulbecco’s Modified Eagle Medium (DMEM) supplemented with 10% heat-inactivated fetal bovine serum, 1% MEM Non-Essential Amino Acids, 1% penicillin/streptomycin and 1% sodium pyruvate. pLVTHM plasmid^59^ encoding either the shRNA sequence 5′-cgcgtGCGCGCTTTGTAGGATTCGttcaagagaCGAATCCTACAAAGCGCGCtttttggaaat -3′ from *Euglena gracilis* chloroplast that does not hybridize with any region of the mouse transcriptome as a negative control or the shRNAs sequences against mouse Crb2:

#1.5′-cgcgtGTGCCAGGCTACAGAAAGTttcaagagaACTTTCTGTAGCCTGGCACtttttggaaat-3′.

#2.5′-cgcgtTGACTTCTACTGCACCTGCttcaagagaGCAGGTGCAGTAGAAGTCAtttttggaaat-3′.

#3.5′-cgcgtCGAAGTGGATGAGGACGAAttcaagagaTTCGTCCTCATCCACTTCGtttttggaaat-3′.

#4.5′-cgcgtCCACCAGAGGAGAGACTTAttcaagagaTAAGTCTCTCCTCTGGTGGtttttggaaat-3′, were transfected into N2A cells with Lipofectamine® LTX and Plus™ Reagent (Life Technologies^TM^) following the manufacturer’s instructions. Cell lysates were collected 36 hours after transfection as described below. Three different experiments were performed and quantified. Data are represented as means ± SEM (Standard Error of the mean).

HEK293 cells were cultured in DMEM supplemented with 10% heat-inactivated fetal bovine serum, 1% MEM Non-Essential Amino Acids, 1% penicillin/streptomycin and 1% sodium pyruvate. pCMV6-AC-GFP plasmid encoding the CRB2 protein with a C-terminal tGFP tag (OriGene Technologies) was transfected into HEK293 cells following the calcium phosphate method, as described in^60^. Total proteins were collected 24 h post-transfection as described below.

### Antibody production

The NCBI database (http://www.ncbi.nlm.nih.gov) was used to choose a unique antigenic amino acid sequence of the mouse CRB2 protein to design an antibody. The sequence of choice of the CRB2 protein, containing amino acids 1243 to 1256 with a cysteine at the N-terminal end, was sent to GenScript Corporation™ for the generation and purification of the corresponding polyclonal antibody.

### Protein extraction and Western blot analyses

The RPE, retina, a portion of brain, a fragment of skeletal muscle, N2A and HEK293 cells in culture were lysed in RIPA buffer (150 mM sodium chloride 1.0% Triton X-100, 0.5% sodium deoxycholate, 0.1% sodium dodecyl sulphate, 50 mM Tris, pH 8.0) with protease inhibitor cocktail (1:1000, Sigma-Aldrich™). Each piece of tissue was homogenized in separate tissue grinders and maintained under constant shaking for 2 h at 4 °C. The resulting solutions were centrifuged at 15,000 g for 20 min at 4 °C. The supernatants were collected and the protein concentrations were assayed with Bradford’s assay, and equal amount of proteins was loaded in each lane.

To discard the possibility that the CRB2 antibody may recognize the CRB3 protein due to aminoacidic similarities, we used 1 · 10^−3^ nmol of CRB3 fused to GST (Abnova) mixed with the same amount of purified BSA (Promega). The later was used to stablish the background threshold of nonspecific binding of the antibody.

Proteins were dissolved in sample buffer (2% SDS, 10% glycerol, 700 mM β-mercaptoethanol, 62.5 mM Tris-HCl, pH 6.8, 0.05% bromophenol blue), and loaded on a SDS-polyacrylamide gel under reducing conditions. After electrophoresis, proteins were transferred to PVDF membranes, blocked for 1 h at RT in a solution with 2% BSA in Tris-buffered saline-Tween (0.1%) (TBST), and immunolabeled overnight at 4 °C with the following primary antibodies: rabbit CRB2 antibody (custom-made, 5 μg/ml), mouse β-actin antibody (Sigma-Aldrich™, 1:5000), mouse GAPDH antibody (Sigma-Aldrich™, 1:10000), rabbit GFP antibody (Clontech, 1:1000), rabbit GST antibody (custom-made^61^, 0.5 μg/ml), mouse PALS1 antibody (Abnova, 1:1000), mouse RPE65 antibody (Abcam™, 1:5000) and mouse Synapsin antibody (Synaptic Systems, 1:1000) in a solution with 2% BSA in TBST. After several washes with TBST, the membranes were incubated with 1:5000 anti-rabbit IgG or 1:30000 anti-mouse IgG conjugated with alkaline phosphatase (Jackson ImmunoResearch™) in 2% BSA, 2% milk in TBST for 60 min at RT, washed with TBST, and developed with NBT (Nitro-blue-tetrazolium, Roche Applied Science™) and BCIP (5-bromo-4-chloro-3-indolyl-phosphate, Roche Applied Science™) or incubated with 1:10000 anti-rabbit IgG, 1:10000 anti-mouse IgG (Jackson ImmunoResearch™) or 1:10000 protein-A (Life Technologies^TM^) conjugated with horseradish peroxidase in 2% BSA and 2% milk in TBST for 60 min at RT, washed with TBST, and developed with Pierce™ ECL Plus Western Blotting Substrate (Thermo Scientific).

We also carried out a peptide competition assay as a negative control. To accomplish this, the CRB2 antibody was incubated with 0.2 mg/ml of the corresponding original antigen fusion protein for 1 h at RT, and this mixture was used instead of the primary antibody dilution.

### Immunofluorescence

To obtain RPE flatmounts, after the animals had been sacrificed their eyes were extracted and the lens and retinas were removed. Then, the RPE and choroid were fixed by immersion for 10 min in PFA 4%. Following this, the fixed tissue was washed in 0.2% PBS-Tx and blocked for 1 h in a solution with 1% BSA and 5% normal serum in 0.2%PBS-Tx and then incubated ON at 4 °C in a solution containing 1% BSA and 2% normal serum in 0.2% PBS-Tx and the following primary antibodies: rabbit CRB2 antibody (custom-made, 5 μg/ml), mouse PALS1 antibody (Abnova, 1:250), Phalloidin (Sigma-Aldrich™, 1:500), mouse p120 catenin antibody (BD Transduction Laboratories, 1:100), mouse RPE65 antibody (Abcam, 1:1000). Then, flatmounts were washed with 0.2% PBS-Tx and incubated for 1 h at RT with 1:500 Alexa fluor 488 and/or 1:500 Alexa fluor 555 fluorescent secondary antibodies (Life Technologies^TM^) and the nuclear-staining TOPRO-3 (Life Technologies^TM^, 1:1000) in 1% BSA and 5% normal serum in 0.2%PBS-Tx. Flatmounts were placed with the choroid facing the slide and the RPE facing the cover slip and mounted using Prolong™ Gold antifading reagent (Life Technologies^TM^).

Negative controls for the flatmounts immunolabeling were performed by removing the primary or secondary antibodies. We also carried out a peptide competition assay, as previously described in the WB analyses, to discriminate any possible background labeling.

### Imaging

All images were obtained with a laser scanning spectral confocal microscope (Leica TCS SP2) with the pinhole set at 1.2 Airy Units and 40x and 63x immersion oil objectives. 488 nm, 543 nm and 633 nm laser lines were used to excite the Alexa 488, Alexa 555 and TOPRO3 fluorochromes respectively, and the three different channels were captured in sequential mode. Minor adjustments of contrast and brightness were performed with Adobe Photoshop CS5 software and Leica Confocal Software. For the 3D reconstructions, thirty-five optical sections were obtained with a voxel depth of 284.9 nm, covering a total depth of 8.8 μm. The image stack obtained was then imported into ImageJ using the LOCI Bio-formats plug-in, and the 3D reconstruction was accomplished using the 3D viewer plug-in for Image J.

## Additional Information

**How to cite this article**: Paniagua, A. E. *et al.* CRB2 completes a fully expressed Crumbs complex in the Retinal Pigment Epithelium. *Sci. Rep.* **5**, 14504; doi: 10.1038/srep14504 (2015).

## Supplementary Material

Supplementary video file
